# EFFECTIVENESS OF A PC EDUCATION PROGRAM IN ASSISTED LIVING IN IMPROVING DISCUSSIONS WITH DEMENTIA FAMILY CAREGIVERS

**DOI:** 10.1093/geroni/igac059.1478

**Published:** 2022-12-20

**Authors:** Debra Dobbs, Jessica Yauk, Carlyn Vogel, Dany Fanfan, Harleah Buck, William Haley, Hongdao Meng

**Affiliations:** University of South Florida, Tampa, Florida, United States; University of South Florida, Tampa, Florida, United States; University of South Florida, Tampa, Florida, United States; University of Florida, Gainesville, Florida, United States; Csomay Center for Gerontological Excellence, Iowa City, Iowa, United States; University of South Florida, Tampa, Florida, United States; University of South Florida, Tampa, Florida, United States

## Abstract

There is a need for improved communication in assisted living communities (ALCs) for staff and family caregivers of persons with dementia. This study is a cluster randomized trial in 10 ALCs (N=23 nurses and administrators; N= 122 residents with dementia) of a PC education (PCEAL) program for dementia care providers facilitated by hospice nurse educators. Baseline, 3- and 6-month data was collected for documentation of advance care planning (ACP) discussions. The PCEAL program had a moderate effect on ACP discussions at the 3-month follow in the treatment (N=17) compared to the control group (N= 6) (Cohen’s d = 0.72, 95% CI [0.25, 1.19]). The findings indicate that the PCEAL program potentially improves ACP discussions with family members of persons with dementia. We will discuss recommendations for tailoring the PCEAL program to include other direct and administrative staff and to elicit the perspective of families about quality of communication.

